# When performance is not enough: elite sport systems during prolonged crises

**DOI:** 10.3389/fspor.2026.1973990

**Published:** 2026-09-23

**Authors:** Olga Kuvaldina

**Affiliations:** 1 Department of Sports, Klaipeda University, Klaipeda, Lithuania; 2 Department of Theoretical Foundation of Olympic and Professional Sports, Admiral Makarov National University of Shipbuilding, Mykolaiv, Ukraine

**Keywords:** crisis, macro-environment, Olympic sport, PEST analysis, Ukraine, war

## 1 Introduction

Elite sport systems operate within increasingly complex environments shaped by interactions between organizational, political, economic, and social factors (1–4). While international sporting success remains the primary objective of most national sport systems, the conditions in which athlete preparation takes place have become progressively less stable. In recent years, elite sport has been affected by global health emergencies, geopolitical instability, economic uncertainty, and armed conflicts, creating challenges that extend beyond the traditional boundaries of sport policy and management (5–7).

The COVID-19 pandemic demonstrated how vulnerable training can be to external disruption. Training opportunities, qualification pathways, international competitions, and athlete mobility were affected across the world (6, 7). Shortly afterwards, the war in Ukraine exposed additional challenges related to security, infrastructure, resource allocation, and the relocation of athletes and coaches (8–11). These events highlighted the extent to which Olympic preparation depends not only on factors within sport systems but also on the broader environment in which they operate. Yet how these external conditions should inform the evaluation of elite sport systems remains underexplored.

Existing elite sport system literature already recognizes the importance of context (3, 4, 12–14). It shows that sporting success is shaped by wider social, political, and economic conditions. However, less attention has been given to evaluation under prolonged crisis conditions. This is especially important when a crisis changes the basic conditions of preparation.

In such situations, the question is not only why a system succeeds in competition. It is also whether the system can preserve or reorganize the conditions required for preparation. This Opinion addresses that gap by proposing a crisis-sensitive approach to evaluating elite sport systems.

The article argues that performance outcomes should remain part of the evaluation. However, medals, rankings, qualification results, and international results should be interpreted alongside external pressures and the system response. In this approach, continuity and resilience become complementary, but distinct, indicators for understanding whether preparation can be sustained under severe disruption.

The Ukrainian Olympic context is used here as an illustrative case, not as a basis for universal generalization. It shows how prolonged crises can shift attention from competitive outcomes alone to the broader policy and organizational factors that allow athletes to continue preparing and competing. The case therefore provides useful context for examining how these factors become visible when disruption is prolonged rather than temporary.

## 2 Beyond performance outcomes

Frameworks such as the Sports Policy Factors Leading to International Sporting Success (SPLISS) and the Global Sporting Arms Race (GSAR) identify financial support, governance, talent development, coaching, sport science, and training infrastructure as important components of elite sport performance. Their strength lies in showing how the organization and resources of sport systems are linked to competitive outcomes. This perspective is particularly useful when comparing national systems and explaining differences in international sporting success (13–15).

The difficulty arises when external disruption changes the environment in which these systems operate. In this Opinion, a prolonged crisis is understood as a disruption that lasts across a substantial part of an Olympic or preparation cycle. It repeatedly affects training, mobility, infrastructure, competition access, or support services. Its importance is not defined by duration alone. It also lies in the inability to return quickly to pre-crisis preparation conditions and in the need to reorganize athlete preparation. Security restrictions, displacement, economic shocks, or limits on mobility may therefore affect preparation independently of coaching quality, talent development, or sport science support. In such circumstances, competitive outcomes alone become harder to interpret as indicators of how well the sport system itself is functioning.

The events of recent years have brought this limitation into sharper focus. COVID-19 (6, 7) and the war in Ukraine (8–11) affected not only sporting outcomes but also the conditions in which those outcomes were produced. In such circumstances, changes in performance may reflect restricted access to training, infrastructure, mobility, or organizational support rather than weaknesses in sport policy alone. This distinction matters because the capacity to sustain preparation may itself become a prerequisite for future sporting success.

During prolonged crises, sustaining preparation means more than keeping sport institutions formally in place. In this Opinion, continuity is treated as an observable state of preparation. It refers to whether essential preparation conditions are maintained, including training, coaching, support services, infrastructure access, mobility, and competition participation. Resilience, by contrast, refers to the dynamic capacity of the sport system to absorb disruption, reorganize resources, adapt preparation routes, and recover over time. This distinction is important because the same degree of continuity may reflect different levels of adaptive capacity depending on the type and severity of external pressure. It is consistent with organizational resilience literature, where resilience is understood as a capacity involving anticipation, coping, adaptation, resources, governance, and organizational learning (16–18). It is also consistent with literature that distinguishes continuity and resilience as related but non-identical concepts (19).

System survival is therefore understood not as institutional existence alone, but as the preservation and reorganization of the minimum conditions required for meaningful athlete preparation during severe disruption.

PEST analysis is commonly used to examine macro-environmental political, economic, socio-cultural, and technological factors that may shape organizational strategy and risk (20). In this Opinion, it is used to identify external pressures that shape Olympic preparation. These pressures may affect training, mobility, infrastructure, support services, and access to competition. PEST does not explain performance outcomes directly. It provides the context for interpreting continuity, resilience, and competitive results. It therefore complements performance-oriented frameworks such as SPLISS and GSAR (13–15).

Figure 1 summarizes the proposed framework by placing performance evaluation within the crisis context in which preparation occurs.

**Figure 1:**
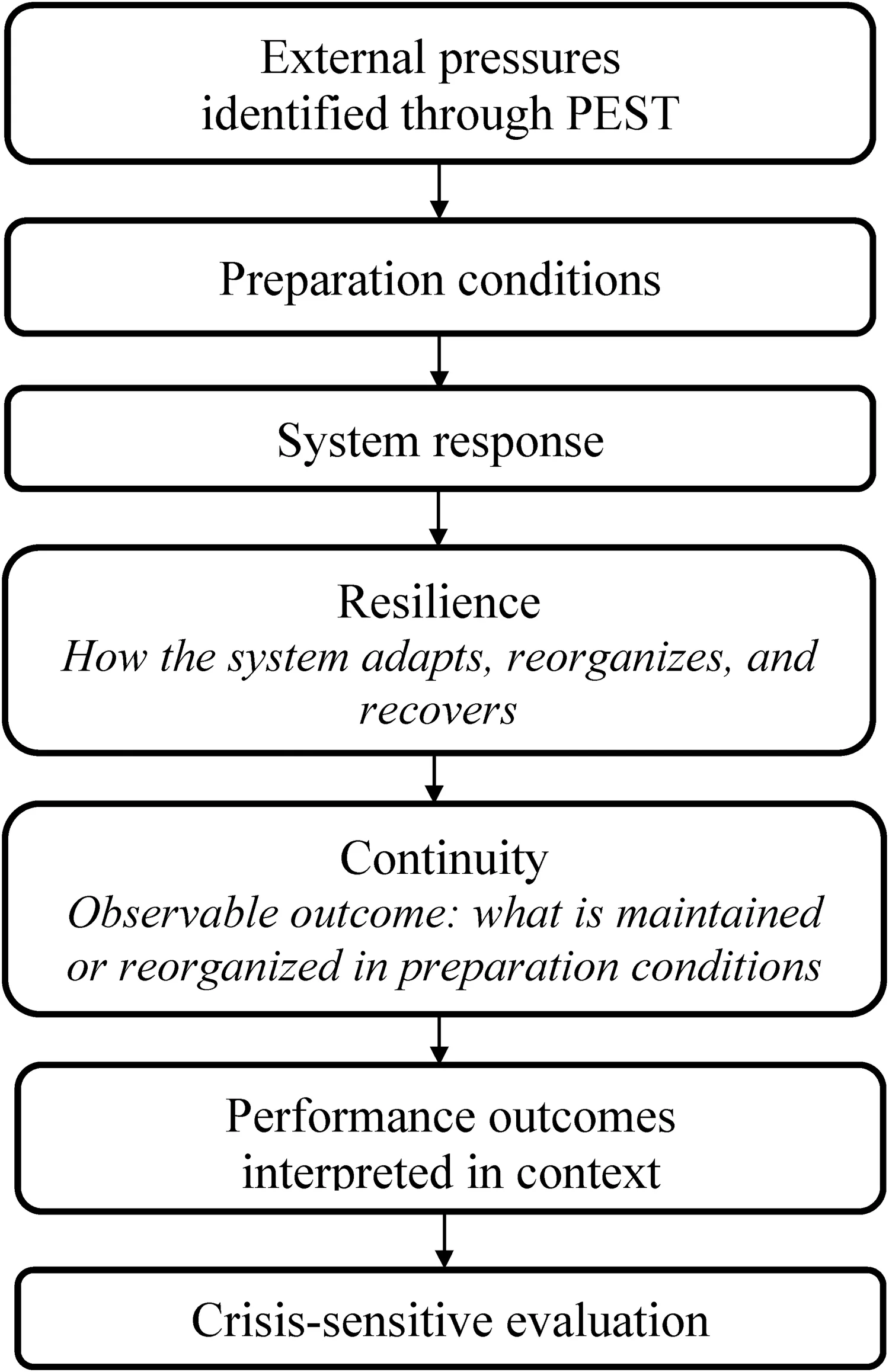
Conceptual structure of crisis-sensitive evaluation of elite sport systems.

A PEST-based approach developed for the Ukrainian Olympic context translated qualitative political, economic, socio-cultural, and technological factors into structured expert ratings, allowing their perceived impact on Olympic preparation to be assessed quantitatively (21). It was subsequently applied to compare the preparation environments of the Ukrainian Olympic team across the Tokyo 2020 and Paris 2024 cycles. These cycles can be understood as Olympic cycles predominantly shaped by different crisis configurations: the COVID-19 pandemic in the Tokyo cycle and the war in Ukraine in the Paris cycle (22). The comparison showed that the dominant external pressures differed between the two cycles: economic and technological factors were more prominent during the Tokyo cycle, whereas political factors became more important during the Paris cycle. Different crises may therefore create different configurations of pressure. Factors that are central in one context may become secondary in another. The external environment is not simply a background condition of elite sport development but part of the preparation context itself, requiring different forms of adaptation even when performance goals remain similar.

The broader implication is that crises do not affect elite sport systems uniformly. Public health emergencies, armed conflict, political instability, sanctions, displacement, or restrictions on international mobility may challenge different parts of the preparation system (23–25).

## 3 Discussion

How, then, should elite sport systems be evaluated when athlete preparation is shaped by forces beyond sport itself? Crises not only interrupt training. They also change the environment in which preparation must be organized and sustained.

Performance indicators such as medals, rankings, qualification outcomes, and international results remain central to elite sport evaluation (13–15). However, during major disruption, they show only part of the picture. A temporary decline in results does not necessarily mean policy failure if organizations are still able to maintain training opportunities, support athletes and coaches, and preserve access to international competition. Conversely, competitive results alone may say little about the adaptive demands faced by systems operating under war, displacement, financial instability, or restricted mobility.

Crisis-sensitive evaluation should therefore include two complementary layers. The first would retain conventional performance indicators. The second layer would examine how the sport system responds to crisis. Continuity would refer to the maintenance of training, coaching, support services, infrastructure access, athlete mobility, and competition participation. Resilience would concern the capacity to reorganize resources, adapt preparation routes, maintain essential organizational functions, and support recovery over time. This approach does not reduce the importance of performance. It recognizes that, during prolonged crises, future performance depends partly on whether the system can preserve the conditions required for preparation.

For sport policymakers, crisis-sensitive evaluation can show which parts of the preparation system are most vulnerable under prolonged disruption. It may help inform decisions on funding, resource allocation, coaching and sport-science support, training infrastructure, international mobility, competition access, and post-crisis recovery. This is especially important when performance results do not fully reflect the constraints under which athletes and sport organizations operate.

Conventional outcomes should therefore be interpreted together with information about whether preparation conditions were maintained under crisis conditions. This may support a more context-sensitive interpretation of whether observed performance changes are plausibly associated with internal system weaknesses, external constraints, or their interaction.

This approach also has limitations. It should not be used to excuse weak governance or remove accountability for competitive outcomes. Nor should resilience become a label for any system that simply continues to operate during crisis. Future research should examine how continuity, understood as the preservation of preparation conditions, and resilience, understood as adaptive capacity, can be assessed and integrated with performance outcomes.
